# Clinical and molecular characterization of giant axonal neuropathy due to a homozygous c.851 + 1G>A variant in *GAN*: A case report and literature review

**DOI:** 10.1016/j.gmg.2026.100098

**Published:** 2026-03-23

**Authors:** Emmanuel Rojas-Morales, Eduardo Esparza-García, María Teresa Magaña-Torres

**Affiliations:** a División de Genética, Centro de Investigación Biomédica de Occidente, Instituto Mexicano del Seguro Social, Sierra Mojada 800, Independencia Oriente, Guadalajara, Jalisco 44340, Mexico; b Doctorado en Genética Humana, Centro Universitario de Ciencias de la Salud, Universidad de Guadalajara, Sierra Mojada 950, Independencia Oriente, Guadalajara, Jalisco 44340, Mexico; c Unidad Médica de Alta Especialidad, Hospital de Pediatría del Centro Médico Nacional de Occidente, Instituto Mexicano del Seguro Social, Belisario Domínguez 735, La Perla, Guadalajara, Jalisco 44360, Mexico

**Keywords:** Giant axonal neuropathy, *GAN*, Whole-exome sequencing, Founder effect, Mexican ancestry

## Abstract

**Background:**

Giant Axonal Neuropathy (GAN) is a rare autosomal recessive neurodegenerative disorder caused by biallelic pathogenic variants in the *GAN* gene, which encodes gigaxonin. The disease typically begins in childhood and is characterized by muscle weakness, gait disturbances, peripheral neuropathy, and distinctive kinky hair.

**Case description:**

We report the clinical, radiological, and molecular findings of a 7-year-old Mexican girl from Jalisco, Mexico, born to consanguineous parents, who presented with global developmental delay and absence of independent ambulation by age of seven. Additional features included kinky hair, broad forehead, blepharoptosis, depressed nasal bridge with a bulbous tip, high-arched palate, micrognathia, and generalized joint hyperlaxity. Given the phenotypic overlap with Noonan syndrome, *PTPN11* Sanger sequencing was initially performed, followed by a RASopathy gene panel; both analyses yielded negative results.

**Results:**

Whole-exome sequencing identified a homozygous splice-site variant in *GAN* c.851 + 1 G>A, which was confirmed by Sanger sequencing. Both parents were heterozygous carriers, consistent with parental consanguinity. According to ACMG/AMP guidelines (PVS1, PM2, PM3), the variant was classified as pathogenic. Additional studies, including brain magnetic resonance imaging, revealed peridentate hyperintensities compatible with gliosis or hypomyelination.

**Conclusion:**

The integration of clinical features, neuroimaging findings, and molecular analysis confirmed the diagnosis of giant axonal neuropathy type 1. This case underscores the clinical relevance of whole-exome sequencing in patients with overlapping syndromic features. Notably, the *GAN* c.851 + 1 G>A variant has previously been reported in at least five homozygous patients of Mexican ancestry, supporting a possible founder effect in this population.

## Highlights

• Homozygous *GAN* c.851 + 1 G>A variant identified in a Mexican patient.
• Noonan syndrome was initially suspected due to overlapping clinical features with GAN.
• Whole-exome sequencing enabled a definitive molecular diagnosis.
• Haplotype analysis supports a possible founder effect.

## Graphical Abstract

**Figure:**
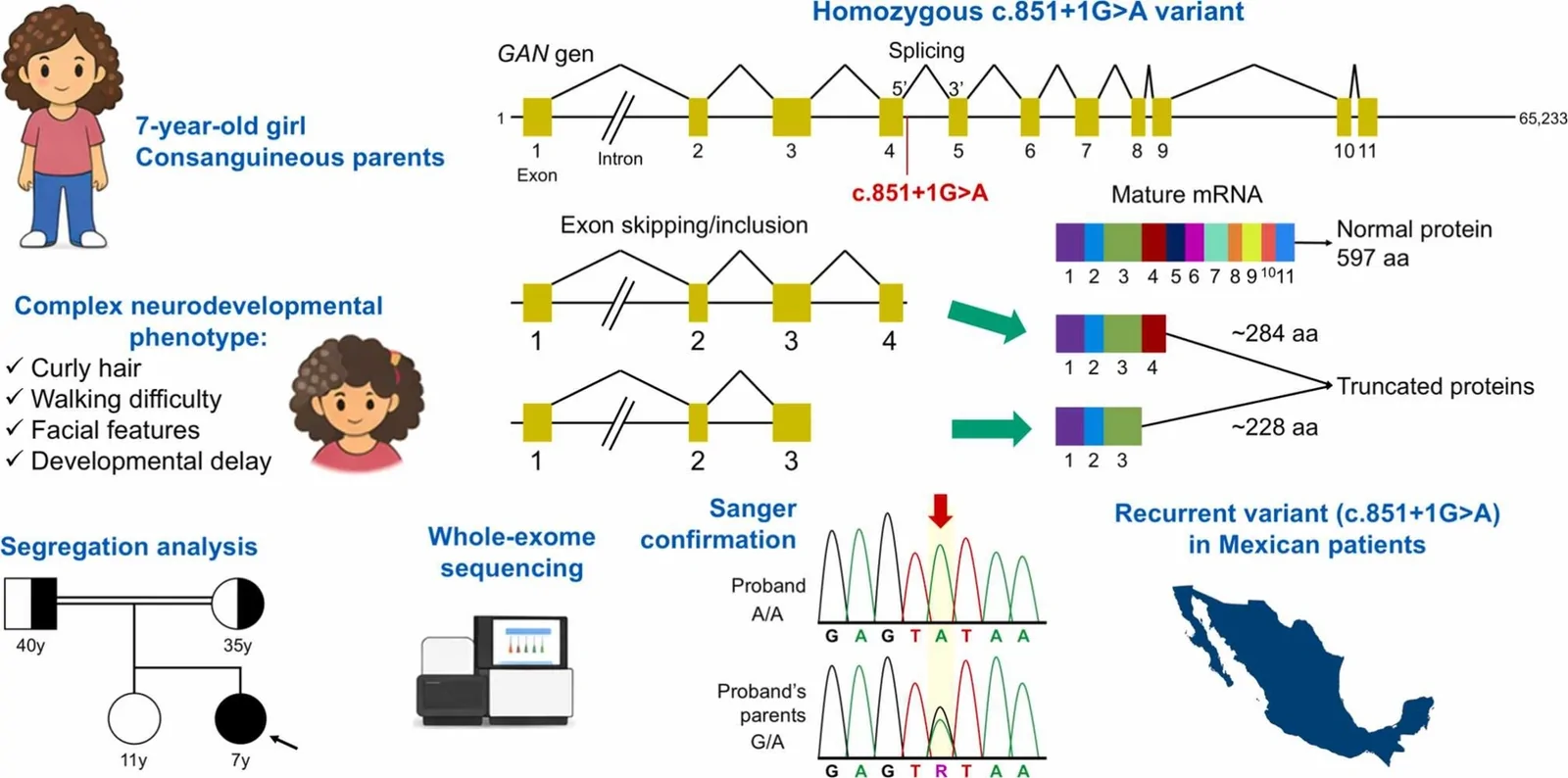

## Introduction

Giant Axonal Neuropathy (GAN; OMIM #256850) is a rare autosomal recessive neurodegenerative disorder caused by biallelic pathogenic variants in the *GAN* gene. The disease is multisystemic and typically begins in childhood, most often presenting with distal muscle weakness, gait disturbances, generalized areflexia, and tightly curled hair. Additional manifestations may include scoliosis, cardiovascular and endocrine involvement, as well as orthopedic, craniofacial, ophthalmological, and skeletal anomalies [1], [2], [3]. Diagnosis of GAN requires integration of clinical assessment with neurophysiological, neuroimaging, and genetic data. Neurophysiological findings usually reveal a progressive axonal sensorimotor neuropathy characterized by reduced compound muscle action potential (CMAPs) and sensory nerve action potentials (SNAPs), affecting both motor and sensory fibers, which helps distinguish GAN from demyelinating neuropathies. Neuroimaging studies may include cerebral white matter abnormalities or cerebellar atrophy [4], [5], [6]. Clinical overlap with hereditary neuropathies (e.g., Charcot–Marie–Tooth; OMIM PS118220) and neurodegenerative disorders with sensory neuropathy (e.g., Friedreich’s ataxia; OMIM #229300), together with the genetic heterogeneity of peripheral neuropathies (>250 genes), makes molecular testing essential [7], [8], [9], [10].

The *GAN* gene (16q23.2) spans 11 exons and encodes gigaxonin, a 597-amino-acid adaptor protein involved in ubiquitin-mediated proteasomal degradation of cytoskeletal proteins such as desmin, MAP1B, and tubulin. Gigaxonin contains an N-terminal BTB domain, a BACK domain, and six C-terminal Kelch repeats [11]; the BTB and BACK domains mediate interaction with Cullin−3 (CUL3) to form a Cullin-RING E3 (CRL3) ubiquitin ligase complex, while the Kelch repeats bind substrate proteins. Loss of gigaxonin function disrupts the ubiquitin–proteasome pathway and autophagy, leading to accumulation of intermediate filaments, axonal disorganization, mitochondrial dysfunction, and progressive neurodegeneration characteristic of GAN [12], [13]. Currently, no curative treatment exists for GAN and management remains primarily symptomatic. Gene therapy has shown partial benefits; however, adverse effects and challenges related to vector delivery continue to represent major obstacles. Future advances will require integrated therapeutic strategies to overcome these barriers and translate experimental findings into meaningful clinical applications [14], [15], [16].

To date, at least 67 causal variants of *GAN* have been reported, including 34 classified as pathogenic, 17 as likely pathogenic, and 16 with potentially or likely pathogenic significance [17], [18], [19]. A recurrent splice-site variant, c.851 + 1 G>A, has been documented in at least five patients of Mexican ancestry who are homozygous for the allele [6]. Here we present the clinical and molecular characterization of an additional patient with giant axonal neuropathy who is homozygous for the pathogenic c.851 + 1 G>A variant in the *GAN* gene.

## Materials and methods

### Ethical considerations

This study was conducted in accordance with the principles of the Declaration of Helsinki and was approved by the research, ethics, and safety committees of our institution. Written informed consent was obtained from all participants prior to the collection of peripheral blood (3–4 mL).

### Molecular analysis

Genomic DNA was extracted from leukocytes using the DTAB/CTAB (dodecyltrimethylammonium bromide–cetyltrimethylammonium bromide) method [20]. Because the proband with suspected Noonan syndrome tested negative for pathogenic variants in *PTPN11* (the gene most commonly altered in this condition) and given that at least 13 genes have been associated with RASopathies [21], with frequent clinical overlap among syndromes, whole-exome sequencing (WES) was pursued to obtain a definitive genetic diagnosis. WES was carried out on a DNBSEQ-G400 system (MGI) by *Gencell-Pharma*, with reported targeting ∼25,000 genes and an average depth of 100X. Primary bioinformatic processing (alignment to the reference genome [GRCh38], variant calling, and quality control) was performed by the service provider. *Variant call files* (VCF) were uploaded to the *Franklin by* QIAGEN platform for annotation and interpretation. The candidate variant (*GAN* c.851 +1 G>A) was confirmed by Sanger sequencing after PCR amplification of a 454 bp fragment encompassing exon 4 and the flanking regions of introns 3 and 4. Primers used were: *forward* 5′-ACTCTGGGAAGTAGACAGTTAGG−3′ and *reverse* 5′-GAACTACCTCTTCCCATACACC−3′. PCR reactions were carried out at a final volume of 10 µL and the amplicons were treated with 0.5 µL of ExoSAP-IT cleanup reagent (*Applied Biosystems, Waltham, MA, USA*). Sequencing reactions were performed with the BigDye Terminator v.3.1 kit (*Applied Biosystems*) according to the manufacturer’s instructions, purified on Sephadex G−50 columns, and analyzed by capillary electrophoresis on a SeqStudio Genetic Analyzer (*Applied Bio-systems*). Electropherograms were reviewed manually and with SnackVar v.2.4.3 using reference sequence NM_022041.4.

## Results

### Clinical presentation

We report a 7-year-old Mexican girl from Tototlán, Jalisco, born to consanguineous parents; her father is also from Tototlán and her mother from Zapotlanejo, Jalisco. She was the product of a second pregnancy and was delivered by cesarean section at 38 weeks of gestation, with a birth weight of 2.8 kg and a length of 49 cm. There were no perinatal complications, and no early feeding or swallowing difficulties were noted. Evaluation by a clinical geneticist documented global developmental delay affecting both motor and language domains. Motor milestones were notably delayed: social smile at 3 months, head control at 6 months, sitting at 8 months, standing with support at 12 months, and assisted ambulation at 18 months. At 7 years of age, she remains unable to walk independently. Language development was also impaired: babbling occurred at 12 months and disyllabic words at 18 months; currently, her expressive vocabulary exceeds 50 words, with simple phrases of up to three words. Bladder and bowel sphincter control has not been achieved. Physical and speech therapy were initiated, with partial improvement.

### Physical examination

On examination, the patient presented normocephaly, kinky hair, a broad forehead, and prominent supraorbital ridges. Facial features included thick eyebrows, horizontal palpebral fissures, telecanthus, depressed nasal bridge with a bulbous tip and thickened alae smooth philtrum, high-arched palate, micrognathia, and pointed chin. The auricles were appropriately positioned but posteriorly rotated, with a poorly defined descending helical fold. The neck was cylindrical, without organomegaly. The thorax and abdomen were symmetric, without structural abnormalities or visceromegaly. External genitalia were female and unremarkable. Limbs and spine displayed symmetrical morphology, and skin color was normal. Generalized joint hyperlaxity was documented, fulfilling Beighton criteria.

### Imaging studies

**Fig. 1:**
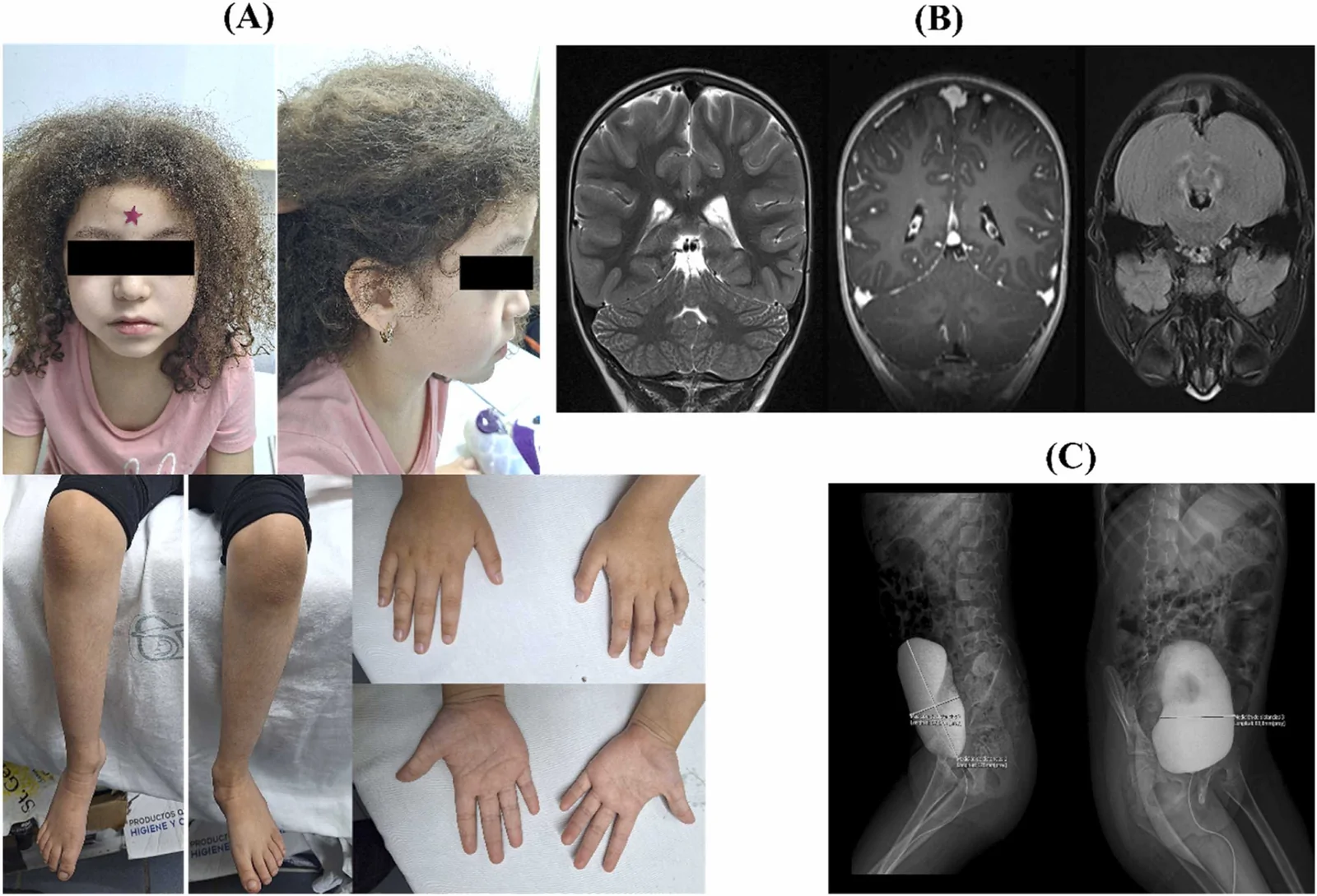
**Clinical and radiologic findings. (A)** Clinical examination shows dysmorphic facial features, kinky hair, bilateral blepharoptosis, small thenar eminence, and reduced distal limb muscle bulk. **(B)** Brain MRI – coronal (top) and axial (bottom) views – demonstrating hyperintense signal in the cerebellar white matter surrounding the dentate nuclei, compatible with gliosis or hypomyelination. **(C)** Contrast cystogram – lateral (left) and anteroposterior (right) projections –showing an enlarged-capacity bladder with extrinsic compression from rectosigmoid contents.

Following the molecular diagnosis, complementary investigations were performed to define the clinical features in greater detail. Re-evaluation confirmed kinky hair, blepharoptosis, and reduced distal limb muscle development (Fig. 1A). Brain magnetic resonance imaging (MRI) (Fig. 1B) revealed hyperintense signal in the cerebellar white matter surrounding the dentate nucleus. These signal abnormalities were interpreted as gliosis or hypomyelination, suggesting either a primary myelination disorder or a chronic neurodegenerative process. The remainder of the brain, including the cerebral hemispheres, brainstem, and basal ganglia, showed normal morphology and preserved volume. The absence of abnormal enhancement after contrast administration ruled out acute inflammatory or neoplastic processes. Abdominal contrast radiography (cystogram, Fig. 1C) showed an increased bladder capacity for age (311 mL). Although markedly distended, the bladder presented regular contours and emptied effectively, with a negligible post-void residual (2%). The main radiologic finding was extrinsic compression of the bladder by abundant rectosigmoid contents, accounting for the megacystis-like appearance rather than an intrinsic outlet obstruction. No vesicoureteral reflux was detected, supporting preserved ureteral function and excluding a common complication of obstructive uropathy.

### Molecular diagnosis

**Fig. 2:**
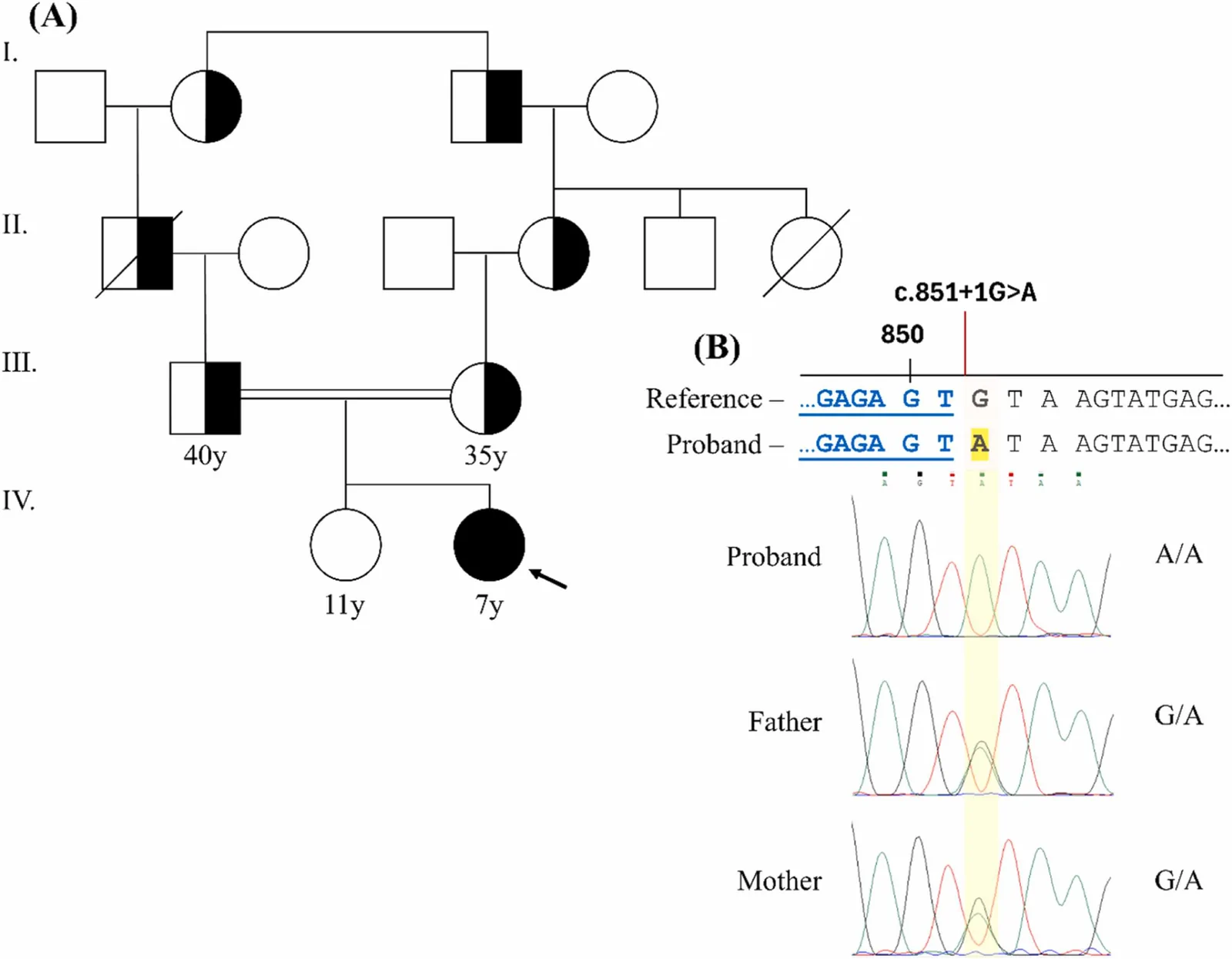
**Segregation analysis and molecular confirmation of the*****GAN*****c.851 + 1 G>A variant. (A)** Pedigree illustrating parental consanguinity, consistent with an autosomal recessive pattern of inheritance. Giant axonal neuropathy is observed in the fourth generation. **(B)** Schematic representation of the *GAN* c.851 + 1 G>A variant affecting the canonical donor splice site at the + 1 intronic position, a region essential for accurate pre-mRNA splicing. Sanger sequencing electropherograms confirm variant segregation within the family.

The initial clinical diagnosis of the proband was Noonan syndrome; therefore, Sanger sequencing of *PTPN11* was performed, and no pathogenic variants were identified. Given the clinical suspicion and the well-recognized genetic heterogeneity of RAsopathies, WES data were subsequently analyzed using a panel of RASopathy-associated genes [21]; however, no pathogenic or likely pathogenic variants were detected. A comprehensive reanalysis of the WES data ultimately revealed a homozygous variant in *GAN*, c.851 + 1 G>A (rs747291494), which was confirmed by Sanger sequencing, thereby establishing the molecular diagnosis. Segregation analysis showed both parents to be heterozygous carriers, and the family reported consanguinity dating back three generations (Fig. 2).

According to ACMG/AMP guidelines [22], the c.851 + 1 G>A variant was classified as pathogenic based on the following criteria: 1) PVS1, as it disrupts the + 1 nucleotide of the canonical splice donor site in intron 4 of the *GAN* gene, for which loss of function is an established disease mechanism; 2) PM2, because the variant is absent or extremely rare in large population databases such as gnomAD (AMR allele frequency = 0.0003581, with no homozygotes reported); and 3) PM3, as the variant was detected in the homozygous state in a patient affected by a recessive disorder, and both parents were confirmed to be heterozygous carriers through segregation analysis.

We assessed whether the c.851 + 1 G>A variant arose once and subsequently spread in the population, by constructing a haplotype across the *GAN* locus. Twelve common variants were genotyped using WES (ordered 5′ to 3′): rs12599564 (c.–323G>T), rs12597517 (c.–235A>G), rs1862843 (c.168–5056 T > C), rs9935709 (c.1236 +256 T > C), rs4889313 (c.1237–59 G>A), rs1037973 (c.1502 +993 A>G), rs1037972 (c.1502 +1044 C>T), rs2216769 (c.*247 G>A), rs1816122 (c.*472 C>G), rs1345895 (c.*592 T > A), rs2550742 (c.*15566 C>G), and rs11412852 (c.*15578_15579insT). We examined the two apparently unrelated carriers available to us—one homozygote (the proband) and one heterozygote—both originating from the same geographic region (Jalisco, Mexico), and found that all three variant-bearing chromosomes (two from the homozygote and one from the heterozygote) shared an identical haplotype 5′-TGCCAGTAGAGT−3′, consistent with a common ancestral origin. These findings support a possible founder effect.

## Discussion

We report a pediatric patient whose phenotype is suggestive of giant axonal neuropathy, with global developmental delay, facial dysmorphism, joint hypermobility, blepharoptosis, reduced muscular development in the limbs, and symmetric hyperintensities in the cerebellar hemispheres [23]. The patient also exhibited less typical features for GAN, including precocious puberty and sleep apnea. Initially, her facial gestalt suggested Noonan-spectrum features, which prompted targeted molecular *PTPN11* testing; however, the result was negative. WES subsequently identified a homozygous splice-site variant, c.851 + 1 G>A, in the *GAN* gene, classified as pathogenic/likely pathogenic [24].

Normendez Martínez et al. reported the first Mexican patient with GAN, a compound heterozygote carrying two missense variants (p.Ala461Val (c.1382 C>T, in exon 9) and p.Arg545Leu (c.1634 G>T, in exon 11)) originating from León, Guanajuato [25]. Both alterations are currently classified as variants of uncertain significance in ClinVar. By contrast, our patient—from Tototlán, Jalisco—harbors the variant c.851 + 1 G>A in the homozygous state. Despite these distinct genotypes, both patients display highly similar, classic clinical features, underscoring a shared core phenotype for the disorder even in the face of molecular heterogeneity. As expected for an autosomal recessive condition, segregation analysis confirmed the biparental origin of the variant, aligning with the reported consanguinity.

*In silico* analysis using PhD-SNPg (score 0.975) [26] predicts loss of the canonical + 1 donor splice site in intron 4, consistent with aberrant splicing that produces a frameshift and premature truncation of gigaxonin. The variant is therefore predicted to generate truncated proteins (∼284 aa from intron 4 retention; ∼228 aa from exon 4 skipping) that retain the BTB and BACK domains but lack other regions essential for normal function, explaining loss-of-function effects and abnormal accumulation of intermediate filaments within axons [23], [27]. Taken together, these computational predictions, the variant’s absence or extreme rarity in population databases, and parental segregation support an ACMG/AMP classification of PVS1 (very strong) + PM2 (moderate) + PM3 (moderate), indicating that the variant is pathogenic and disease-causing.

The recurrence of c.851 + 1 G>A among patients of Mexican ancestry—our case plus five previously reported non-consanguineous homozygotes [6]— raises the possibility of a founder effect. Genotyping of two apparently unrelated carriers revealed the same haplotype, 5′-TGCCAGTAGAGT−3′, linked to the c.851 + 1 G>A variant, consistent with a shared ancestral origin. However, definitive confirmation requires population-level allele frequencies and extended haplotype larger and more geographically diverse samples to distinguish a single founder event from recurrent independent mutations or a mutational hotspot. If a founder origin is demonstrated, the finding would support targeted carrier screening and diagnostic prioritization in Mexican cohorts, providing grounds to inform genetic counseling as well as public health strategies.

To date, more than 500 variants in *GAN* have been reported in the literature and classified as variants of uncertain significance, likely pathogenic, or pathogenic. Although missense variants predominate (356 variants), robust evidence of pathogenicity is available for only 18. As shown in Appendix A (Fig. S1), these variants cluster within key functional regions of gigaxonin, including the BTB and BACK domains, as well as at splice sites in the *GAN* gene. This pattern is consistent with a loss-of-function disease mechanism, in which truncating alleles generally provide stronger evidence of pathogenicity than missense variants. Thus far, 13 canonical splice-site variants have been reported in *GAN* [25].

Table 1 summarizes the clinical features of twelve patients carrying one of three splice-site variants: c.851 + 1 G>A, c.1374 + 1 G>A (predicted to generate two isoforms 465 and 438 aa), and c.1502 + 1 G>T (551 and 555 aa). Across these cases, a consistent core phenotype was observed, including kinky hair, areflexia, distal lower-limb weakness, and peripheral neuropathy. Notably, patients harboring c.1502 + 1 G>T (reported in Turkish and Syrian cohorts) also manifested additional cerebellar features—nystagmus, dysmetria, and radiological cerebellar atrophy— raising the possibility of a broader clinical spectrum for this particular allele [6], [28], [29], [30], [31]. These phenotypic differences might reflect variant-specific effects on the repertoire and length of truncated gigaxonin isoforms, but they might just as well be influenced by modifying variants in other genes or by non-genetic factors.

**Table 1:** Clinical and Molecular features of Published splice-site variants in the *GAN* gene.

**Variant**	**Population**	**Origin**	**Type of study**	**Clinical characteristics**	**Method**	**Reference**
c.851 + 1 G>A	1 Patient - 7-year-old girl	Mexican	Report	Global developmental delay, facial dysmorphic features, kinky hair, broad forehead, prominent supraciliary arches, thick eyebrows, horizontal palpebral fissures, telecanthus, depressed nasal bridge, bulbous nose with thickened alae, flattened philtrum, high palate, micrognathia, pointed chin, adequately set auricles but posteriorly rotate, poorly defined downward helical fold, joint hypermobility (positive Beighton criteria).	WES	In this study
	5 Patients (non- consanguineous)	Mexican descent	Cohort	Macrocephaly, kinky hair, dysarthria, decreased visual acuity, urinary tract abnormalities, precocious puberty, sleep apnea.	NS	[6]
	1 Patient - 6-year-old boy	NS	Cohort	Progressive gait disturbance, hammer toes, pes cavus, lower limb areflexia, abnormal brain morphology.	WES	[28]
c.1373 + 1 G>A	1 Patient - 8-year-old girl	China	Report	Thick, kinky hair; muscle weakness in lower limbs (MRC grade 4/5); bilateral Achilles tendon contractures, absent knee and Achilles tendon reflexes, distal muscle atrophy, axonal sensorimotor neuropathy on nerve conduction studies and electromyography, abnormal signal in both dentate nuclei on MRI.	WES	[29]
c.1502 + 1 G>T	2 Patients – 14-year-old girl 15-year-old boy	Turkish	Report	Areflexia, kinky hair, nystagmus, dysmetria, cerebellar atrophy.	Sanger	[30]
	2 Families (non- consanguineous)	Turkish	Cohort	Peripheral neuropathy, cerebellar ataxia, frizzly hair, scoliosis, Babinski’s sign positive.	Sanger	[31]

Note: NS = Not specified. WES= Whole-exome sequencing

The main limitation of this study lies in the inclusion of a single case, lacking population-based data to assess the regional frequency of the variant.

## Conclusion

The integration of clinical, neuroimaging, and molecular findings confirmed the diagnosis of giant axonal neuropathy type 1 in a patient harboring the homozygous splice-site variant *GAN*, c.851 + 1 G>A, a pathogenic loss-of-function allele consistent with the classical phenotype. This case underscores the diagnostic value of whole-exome sequencing in patients with overlapping clinical presentations and raises the possibility of a founder effect for this variant, a hypothesis that warrants further investigation.

## Ethical Approval

The study was conducted in accordance with the Declaration of Helsinki and approved by both the Research and Ethics Committees at our *Centro de Investigación Biomédica de Occidente-IMSS* (CONBIOETICA −09-CEI−009–20160601) (approval date: 23 June 2021; protocol number R−2021–785–068).

## Funding

This work was supported by 10.13039/501100004881Instituto Mexicano del Seguro Social (IMSS) (grant number R−2021–785–068). The funding source had no role in study design, data analysis, interpretation, or manuscript preparation.

## CRediT authorship contribution statement

Conceptualization, M.T.M.-T.; methodology, M.T.M.-T.; formal analysis, E.R.M., and M.T.M.-T.; investigation, E.R.M., M.T.M.-T., and E.E.-G.; resources, M.T.M.-T.; writing—original draft preparation, E.R.M., M.T.M.-T., and E.E.-G.; writing—review and editing, E.R.M., and M.T.M.-T.; supervision, M.T.M.-T.; project administration, M.T.M.-T.; funding acquisition, M.T.M.-T. All authors have read and agreed to the published version of the manuscript.

## Informed consent

Written informed consent was obtained from all participants prior to sample collection.

## Declaration of Competing Interest

The authors declare no conflicts of interest.

## Data Availability

Data supporting the findings of this study are available within the article and its supplementary material. Additional data are available from the corresponding author upon reasonable request.

## Footnotes

Appendix A Supplementary data associated with this article can be found in the online version at doi:10.1016/j.gmg.2026.100098.

## References

[1] Kang J.J., Liu I.Y., Wang M.B., Srivatsan E.S. (2016). A review of gigaxonin mutations in giant axonal neuropathy (GAN) and cancer. Hum. Genet.

[2] Garg M., Kulkarni S.D., Udwadia Hegde A., Desai M., Sayed R.J. (2018). Giant axonal neuropathy: Clinical, radiological, and genetic features. Ann. Indian Acad. Neurol..

[3] Gargano M.A., Matentzoglu N., Coleman B., Addo Lartey E.B., Anagnostopoulos A.V., Anderton J., Avillach P., Bagley A.M., Bakštein E., Balhoff J.P. (2024). The Human Phenotype Ontology in 2024: Phenotypes around the world. Nucleic Acids Res.

[4] Lehmann H.C., Wunderlich G., Fink G.R., Sommer C. (2020). Diagnosis of peripheral neuropathy. Neurol. Res. Pr..

[5] Xu X., Yang X., Su Z., Wang H., Li X., Sun C., Wang W., Chen Y., Zhang C., Zhang H. (2020). Identification of novel compound heterozygous mutations in the GAN gene of a Chinese patient diagnosed with giant axonal neuropathy. Front. Neurosci..

[6] Bharucha Goebel D.X., Norato G., Saade D., Paredes E., Biancavilla V., Donkervoort S., Kaur R., Lehky T., Fink M., Armao D. (2021). Giant axonal neuropathy: Cross-sectional analysis of a large natural history cohort. Brain.

[7] Szigeti K., Lupski J.R. (2009). Charcot-Marie-Tooth disease. Eur. J. Hum. Genet.

[8] Nagappa M., Sharma S., Taly A.B. (2024). Charcot-Marie-Tooth disease. StatPearls.

[9] Mahale R., Purushottam M., Singh R., Yelamanchi R., Kamble N., Holla V., Pal P.K., Jain S., Yadav R. (2024). Revisiting Friedreich’s ataxia: Phenotypic and imaging characteristics. Ann. Indian Acad. Neurol..

[10] Rossor A.M., Haddad S., Reilly M.M. (2024). The evolving spectrum of complex inherited neuropathies. Curr. Opin. Neurol..

[11] Lescouzères L., Bomont P. (2020). E3 ubiquitin ligases in neurological diseases: Focus on gigaxonin and autophagy. Front. Physiol..

[12] Wang W., Ding J., Allen E., Zhu P., Zhang L., Vogel H., Yang Y. (2005). Gigaxonin interacts with tubulin folding cofactor B and controls its degradation through the ubiquitin-proteasome pathway. Curr. Biol..

[13] Shirakaki S., Roshmi R.R., Yokota T. (2023). Genetic approaches for the treatment of giant axonal neuropathy. J. Pers. Med..

[14] Bailey R.M., Armao D., Nagabhushan Kalburgi S., Gray S.J. (2018). Development of intrathecal AAV9 gene therapy for giant axonal neuropathy. Mol. Ther. Methods Clin. Dev..

[15] Armao D., Bouldin T.W., Bailey R.M., Gray S.J. (2021). Extensive rod and cone photoreceptor-cell degeneration in rat models of giant axonal neuropathy: Implications for gene therapy of human disease. Ophthalmic Genet.

[16] Bharucha Goebel D.X., Todd J.J., Saade D., Norato G., Jain M., Lehky T., Bailey R.M., Chichester J.A., Calcedo R., Armao D. (2024). Intrathecal gene therapy for giant axonal neuropathy. N. Engl. J. Med..

[17] Miller M.P., Kumar S. (2001). Understanding human disease mutations through the use of interspecific genetic variation. Hum. Mol. Genet.

[18] David A., Sternberg M.J. (2015). The contribution of missense mutations in core and rim residues of protein-protein interfaces to human disease. J. Mol. Biol..

[19] Nesta A.V., Tafur D., Beck C.R. (2021). Hotspots of human mutation. Trends Genet.

[20] Gustincich S., Manfioletti G., Del Sal G., Schneider C., Carninci P. (1991). A fast method for high-quality genomic DNA extraction from whole human blood. BioTechniques.

[21] Rehm H.L., Berg J.S., Brooks L.D., Bustamante C.D., Evans J.P., Landrum M.J., Ledbetter D.H., Maglott D.R., Martin C.L., Nussbaum R.L. (2015). ClinGen—the Clinical Genome Resource. N. Engl. J. Med..

[22] Richards S., Aziz N., Bale S., Bick D., Das S., Gastier-Foster J., Grody W.W., Hegde M., Lyon E., Spector E., Voelkerding K., Rehm H.L., ACMG Laboratory Quality Assurance Committee (2015). Standards and guidelines for the interpretation of sequence variants: A joint consensus recommendation of the American College of Medical Genetics and Genomics and the Association for Molecular Pathology. Genet. Med..

[23] Gangfuß A., Goj G., Polz S., Della Marina A., Hentschel A., Ahlbory K., Deba T., Kotzaeridou U., Schuler E., Pechmann A. (2025). Giant axonal neuropathy (GAN): Cross-sectional data on phenotypes, genotypes, and proteomic signature from a German cohort. J. Neurol..

[24] Landrum M.J., Chitipiralla S., Kaur K., Brown G., Chen C., Hart J., Hoffman D., Jang W., Liu C., Maddipatla Z. (2025). ClinVar: Updates to support classifications of both germline and somatic variants. Nucleic Acids Res.

[25] Normendez Martínez M.I., Monterde Cruz L., Martínez R., Marquez Harper M., Esquitin Garduño N., Valdes Flores M., Casas Avila L., Ponce de Leon-Suarez V., Romero Díaz V.J. (2018). Two novel mutations in the GAN gene causing giant axonal neuropathy. World J. Pedia.

[26] Capriotti E., Fariselli P. (2023). PhD‑SNPg: updating a webserver and lightweight tool for scoring nucleotide variants. Nucleic Acids Res.

[27] Renganathan B., Zewe J.P., Cheng Y., Paumier J.M., Kittisopikul M., Ridge K.M., Opal P., Gelfand V.I. (2023). Gigaxonin is required for intermediate filament transport. FASEB J..

[28] Chakravorty S., Logan R., Elson M.J., Luke R.R., Verma S. (2020). Expanding the genotype-phenotype correlation of childhood sensory polyneuropathy of genetic origin. Sci. Rep..

[29] Guo Y., Su Q., Zhu X., Wang J., Lou Y., Miao P., Wang Y., Zhang B., Jin Y., Gao L. (2022). Giant axonal neuropathy (GAN) in an 8-year-old girl caused by a homozygous pathogenic splicing variant in GAN gene. Am. J. Med. Genet. A.

[30] Incecik F., Herguner O.M., Ceylaner S., Zorludemir S., Altunbasak S. (2015). Giant axonal disease: Report of eight cases. Brain Dev..

[31] Demir E., Bomont P., Erdem S., Cavalier L., Demirci M., Kose G., Muftuoglu S., Cakar A.N., Tan E., Aysun S. (2005). Giant axonal neuropathy: Clinical and genetic study in six cases. J. Neurol. Neurosurg. Psychiatry.

