# Supplementary material for: Clinical and molecular characterization of giant axonal neuropathy due to a homozygous c.851 + 1G>A variant in GAN: A case report and literature review
Source: Glob Med Genet. 2026 Mar 23;13(3):100098. doi: 10.1016/j.gmg.2026.100098 (PMC13562385; doi:10.1016/j.gmg.2026.100098)
Supplement: Supplementary file 1 [file mmc1.docx]

**Appendix A**

**Fig. S1. Pathogenic and likely pathogenic variants reported in *GAN*.** In the upper section **(A)**, the gene structure is shown, with its 11 exons, and the location of pathogenic (Red), likely pathogenic (Blue) and Uncertain significance (green) variants reported in the literature and databases. The lower section **(B)** represents the architecture of the gigaxonin protein (597 amino acids), detailing its key functional domains: BTB, BACK, and the six *Kelch* repeats. Furthermore, the regions where variants that directly impact the protein's structure and/or function are concentrated are indicated. Section **(C)** shows the distribution of exons along the protein.
